# Assessment of hepatic fibrosis with non-invasive indices in subjects with diabetes before and after liver transplantation

**DOI:** 10.3389/fendo.2024.1359960

**Published:** 2024-03-05

**Authors:** Valeria Grancini, Irene Cogliati, Gianfranco Alicandro, Alessia Gaglio, Stefano Gatti, Maria Francesca Donato, Emanuela Orsi, Veronica Resi

**Affiliations:** ^1^ Endocrinology Unit, Fondazione IRCCS Ca’ Granda Ospedale Maggiore Policlinico, Milan, Italy; ^2^ Department of Pathophysiology and Transplantation, Università degli Studi di Milano, Milan, Italy; ^3^ Department of Pediatrics, Cystic Fibrosis Center, Fondazione IRCCS Ca’ Granda Ospedale Maggiore Policlinico, Milan, Italy; ^4^ Center for Preclinical Research, Fondazione IRCCS Ca’ Granda - Ospedale Maggiore Policlinico, Milan, Italy; ^5^ Hepatology Unit, Fondazione IRCCS Ca’ Granda Ospedale Maggiore Policlinico, Milan, Italy

**Keywords:** diabetes mellitus, liver transplantation, fibrosis, APRI score, FIB-4

## Abstract

**Introduction:**

One of the most common complications of cirrhosis is diabetes, which prevalence is strictly related to severity of hepatopathy. Actually, there are no data on the persistence of post-transplant glucose abnormalities and on a potential impact of diabetes on development of fibrosis in the transplanted liver. To this aim, we evaluated liver fibrosis in cirrhotic subjects before and after being transplanted.

**Methods:**

The study included 111 individuals who had liver transplantation. The assessment was performed before and two years after surgery to investigate a potential impact of the persistence of diabetes on developing *de novo* fibrosis in the transplanted liver. The degree of fibrosis was assessed using the Fibrosis Index Based on 4 Factors (FIB-4) and the Aspartate to Platelet Ratio Index (APRI).

**Results:**

At pre-transplant evaluation, 63 out of 111 (56.8%) subjects were diabetic. Diabetic subjects had higher FIB-4 (Geometric mean, 95% confidence interval: 9.74, 8.32-11.41 *vs* 5.93, 4.71-7.46, *P*<0.001) and APRI (2.04, 1.69-2.47 *vs* 1.18, 0.90-1.55, *P*<0.001) compared to non-diabetic subjects. Two years after transplantation, 39 out of 111 (35.1%) subjects remained with diabetes and continued to show significantly higher FIB-4 (3.14, 2.57-3.82 *vs* 1.87, 1.55-2.27, *P*<0.001) and APRI (0.52, 0.39-0.69 *vs* 0.26, 0.21-0.32, *P*<0.001) compared to subjects without diabetes.

**Discussion:**

Thus, persistence of diabetes after surgery is a possible risk factor for an evolution to fibrosis in the transplanted liver, potentially leading to worsened long-term outcomes in this population.

## 1 Introduction

Actually, liver biopsy is considered the gold standard to assess liver fibrosis (1, 2). However, the widespread use of this procedure to determinate the degree of liver fibrosis in everyday practice is hardly feasible for several reasons. The procedure is costly and invasive, causing discomfort, pain and potential serious complications, as bleeding and, although rare, even death (3–6). Moreover, a considerable variability in sampling and in the histopatological interpretation has been reported, leading to possible underestimation of the stage of fibrosis (7, 8).

Transient elastography (FibroScan) has been proposed by the “European Association for the Study of the Liver” (EASL) and the “American Association for the Study of Liver Diseases” (AASLD) for the assessment of hepatic fibrosis in individuals with non-alcoholic fatty liver disease (NAFLD). Therefore, FibroScan is currently the most widely used and validated alternative to liver biopsy (9–12). Its value lies in its relatively inexpensive cost and portability, but this method can be considerably limited by obesity (11–13) and it is rarely available in the context of a diabetes outpatient visit.

Apart from FibroScan, over the past decade, other potential less-invasive techniques have been proposed for the evaluation of hepatic fibrosis, and their concordance with liver biopsy results has been demonstrated in different populations, especially in people with viral hepatitis and NAFLD. The most widely used are the “aspartate aminotransferase (AST) to alanine aminotransferase (ALT) ratio” (13), the “age-platelet index (14), the aspartate aminotransferase to platelet ratio index” (APRI) (15) and the “Fibrosis Index Based on 4 Factors” (FIB-4) (16). In the “Edinburgh type 2 diabetes study”, Morling JR et al. demonstrated that the APRI and FIB-4 had the best positive agreement in detecting the presence of liver fibrosis in individuals with type 2 diabetes mellitus (17). Moreover, Ciardullo S et al. recently validated the use of non-invasive scores (in particular age-adjusted FIB-4) among a wide population of individuals with diabetes to characterize subjects at risk for fibrosis, making referrals to hepatologist more sustainable (18). Again, Ciardullo S et al. showed also that the screening for hepatopathy in a population of individuals with diabetes utilizing a combination of imaging-based techniques and serum-based indexes could reduce the need for hepatic biopsy (19). Finally, Kitajima T. et al. validated the FIB-4 for assessment of fibrosis in subjects who have undergone liver transplantation (20).

Although elastography overcomes the surrogate indexes for identify people at risk for fibrosis, serum markers have greater feasibility, being they are simpler, more reproducible and accessible with good reliability (9).

Type 2 diabetes mellitus is a very common condition in people with hepatopathy, and the relationship between these two conditions is bidirectional (21, 22): the contribution of cirrhosis to development of alterations in glucose metabolism has been widely demonstrated; conversely, diabetes can accelerate the progression to severe hepatopathy (23). Today, the real contribution of diabetes in developing and worsening liver disease is still debated (24), but a plenty of literature is available on the strong bond between diabetes, insulin resistance, plasma glucose and hepatic fibrosis in individuals with hepatopathy, and these evidences come primarily from HCV-infected subjects (25–31).

Currently there is no exhaustive evidence on the influence of diabetes on hepatic fibrosis progression after transplantation. For this reason, we calculated APRI and FIB-4 in people with cirrhosis referring to our Diabetology Unit, who have undergone liver transplantation, to assess the impact of diabetes on hepatic fibrosis progression before and after liver transplantation.

## 2 Research design and methods

### 2.1 Study design

We conducted an observational, prospective study aimed at assessing the relationship between diabetes and liver fibrosis in individuals with cirrhosis who underwent liver transplantation. The study complies with the Declaration of Helsinki. The research protocol was approved by the Ethics Committee of the IRCCS Cà Granda – Ospedale Maggiore Policlinico Foundation (Prot. n. 516) and written informed consent was provided by each participant.

### 2.2 Patient population

From January 2014 to December 2018, 187 consecutive subjects with liver cirrhosis, who were candidates to liver Tx, were evaluated at our Endocrinology Unit. Of them, 111 individuals underwent transplantation (sex, according to SAGER guidelines – 32: 81 males/30 females), completed a 2-year follow-up and were included in this analysis.

### 2.3 Measurements

At enrolment, a complete medical history was collected for each patient. Before and two years after transplantation all individuals had an anthropometric assessment and clinical parameters were recorded. Furthermore, all patients had a fasting blood sample to evaluate glycaemic control and hepatic function. Both at enrolment and 24 months after surgery, subjects underwent a 75 g OGTT to diagnose diabetes according to the American Diabetes Association criteria (32).

Based on body mass index (BMI) values, they were classified as underweight (<18 kg/m^2^), normal weight (18.5-24.9 kg/m^2^), overweight (25-29.9 kg/m^2^) or obese (≥30 kg/m^2^).

### 2.4 Calculation of liver fibrosis indices

APRI was calculated as AST/(upper limit of the normal range) x 100/platelet counts (PLT) (10^9^/L) (15),. FIB-4 was calculated as age (years) x AST (IU/L)/(PLT [10^9^/L] x ALT [IU/L]^1/2^) (16),.

### 2.5 Statistical analysis

Categorical variables were summarized as frequencies and percentages, whereas continuous variables as median (25^th^-75^th^ percentile). Differences in baseline characteristics between people without or with diabetes were compared using the Fisher’s exact test, the Chi-square test or the Wilcoxon rank sum test according to the type of variable and frequency count.

The prevalence of diabetes was calculated by dividing the number of diagnosed subjects by the total number of enrolled people, and 95% confidence intervals were calculated using the binomial distribution.

To account for the positive skewness in the data and to reduce the influence of outliers, data on liver fibrosis markers were summarized using the geometric mean and corresponding 95% confidence interval.

Linear mixed-effects models with random intercept were used to evaluate the effects of diabetes status (as time varying covariate) and timing of measurement (pre vs post transplantation) on the FIB-4 and APRI. The models included the indices of liver fibrosis as response variables, main effects for diabetes status and time, a diabetes-by-time interaction term and age at measurement as covariate. Response variables were included in the models as natural log transformed variables. Model-based means were then back-transformed, to geometric means in the original scale. Beta coefficients were exponentiated to represent ratios of geometric means. Metabolic risk factors including changes in weight and BMI as well as fasting glycaemia, HbA1c, serum creatinine, total cholesterol, LDL, HDL and triglycerides were compared between patients who had diabetes post transplantation and those who did not using the Wilcoxon rank sum test, with *P* values adjusted for multiple testing. Statistical significance was determined by *P* values < 0.05.

## 3 Results

The study included 111 individuals. Based on the results of the OGTT performed before transplantation, subjects were classified into glucose tolerance categories as follows: 63 as with diabetes, 23 with impaired glucose tolerance (IGT), four with impaired fasting glucose (IFG), two patients with both IFG and IGT, and 19 with normal glucose tolerance (NGT). The prevalence of diabetes among these subjects was 56.8% (95% CI: 47.0-66.1).

**Table 1** shows a comparison of baseline characteristics between the 48 people without diabetes and the 63 ones with diabetes. subjects with diabetes were older (median age 58 vs 51.5), had higher levels of AST, ALT as well as higher values of FIB-4 and APRI. Platelet count was lower in people with diabetes as compared to individuals without diabetes. BMI was not significantly different between groups. Around half of the subjects were overweight or obese with no statistically significant differences between groups. In approximately 75% of the cases, viral hepatitis was identified as the primary cause of cirrhosis with no significant differences between the two subpopulations. Liver disease duration were comparable between groups.

**Table 1:** Population Characteristics.

Characteristic	Non-diabetic patients, N = 48^1^	Patients with diabetes, N = 63^1^	*P* value^2^
Female Sex (33)	11 (22.9%)	19 (30.2%)	0.395
Age	51.5 (46.0, 59.0)	58.0 (52.5, 61.0)	0.005
BMI (kg/m^2^)	24.7 (23.5, 28.2)	25.5 (23.2, 28.5)	0.732
BMI category			0.745
Underweight	0	0	
Normal weight	25 (52.1%)	29 (46.0%)	
Overweight	18 (37.5%)	25 (39.7%)	
Obesity	5 (10.4%)	9 (14.3%)	
Duration of liver disease	10.0 (3.0, 19.0)	9.0 (4.0, 18.5)	0.898
Etiology of liver disease			0.156
Autoimmune disease	3 (6.3%)	3 (4.8%)	
Alcohol	7 (14.6%)	7 (11.1%)	
HBV	3 (6.3%)	6 (9.5%)	
HCV	15 (31.3%)	34 (54.0%)	
HBV + HCV	3 (6.3%)	4 (6.3%)	
HBV + HDV	8 (16.7%)	4 (6.3%)	
Other	9 (18.8%)	5 (7.9%)	
Hepatocellular carcinoma	17 (35.4%)	32 (50.8%)	0.106
AST	55.5 (33.8, 87.3)	75.0 (48.5, 110.0)	0.019
ALT	36.5 (20.8, 52.3)	47.0 (31.0, 67.5)	0.019
GGT	59.5 (39.8, 114.5)	61.0 (36.5, 108.0)	0.981
Fasting glycaemia (mg/dL)	86.0 (82.0, 94.3)	103.0 (91.0, 121.0)	<0.001
Glucose tolerance category			
Diabetes	0	63 (100.0%)	
IFG	4 (8.3%)	0	
IFG+IGT	2 (4.2%)	0	
IGT	23 (47.9%)	0	
NGT	19 (39.6%)	0	
Platelet count (x 10^9^/L)	71.5 (54.0, 109.8)	62.0 (46.5, 86.0)	0.032
FIB-4	7.0 (3.8, 10.9)	9.7 (6.4, 14.5)	0.002
APRI	1.5 (0.6, 2.1)	2.0 (1.2, 3.2)	0.005

^1^n (%); Median (25th -75th percentile).

^2^Pearson’s Chi-squared test; Wilcoxon rank sum test; Fisher’s exact test.

ALT, Alanine transaminase. APRI, Aspartate aminotransferase to platelet ratio index. AST, Aspartate aminotransferase. BMI, Body mass index. FIB-4, Fibrosis Index Based on 4 Factors. GGT, Gamma-glutamyl transferase. HBV, Hepatitis B virus. HCV, Hepatitis C virus. HDV, hepatitis D virus. IFG, Impaired fasting glucose. IGT, Impaired glucose tolerance. NGT, Normal glucose tolerance.

At the two-year follow-up visit, 41 individuals had an OGTT indicative of diabetes resulting in a prevalence of 36.9% (95% CI: 28.0-46.6). Additionally, 12 subjects were classified as IGT, six as having IFG, 10 with both IFG and IGT and 42 as NGT. Out of the 48 who were non-diabetic prior to liver transplantation, three patients developed diabetes after transplantation.

Regarding to immunosuppressant therapy, subjects from both groups were placed on steroid therapy in the immediate post-surgery period and, after that, prednisone was gradually decreased (until suspended) and combined with calcineurin inhibitors (tacrolimus in most cases).

**Table 2** summarizes the FIB-4 and the APRI values according to diabetes status and time from liver transplantation. Post-transplantation values were lower compared to those observed 2 years after liver transplantation. Having a diagnosis of diabetes is associated with higher FIB-4 and APRI values at both time points.

**Table 2:** Markers of liver fibrosis according to diabetes status and time from liver transplantation.

Marker of liver fibrosis	Non-diabetic patients		Patients with diabetes	
	Pre-Tx, N= 48	Post-Tx, N= 72	Pre-Tx, N= 63	Post-Tx, N= 39
FIB-4	5.93 (4.71-7.46)	1.87 (1.55-2.27)	9.74 (8.32-11.41)	3.14 (2.57-3.82)
APRI	1.18 (0.90-1.55)	0.26 (0.21-0.32)	2.04 (1.69-2.47)	0.52 (0.39-0.69)

Data are geometric means (95% confidence intervals).

APRI, Aspartate aminotransferase to platelet ratio index. FIB-4, Fibrosis Index Based on 4 Factors. Tx, Liver transplantation.

**Table 3** shows the results of the regression models. Having diabetes was associated with a 48% (95% CI: 21-83) higher geometric mean for FIB-4 and 78% (38-129%) higher geometric mean for APRI as compared to not being diagnosed with diabetes. The geometric means observed after liver transplantation were 70% (95% 65-75) lower for FIB-4 and 66% (71–81) lower for APRI than those recorded before liver transplantation. The reduction was not significant different between non-diabetic and diabetic patients (*P* values for the interaction term: 0.70 for FIB-4 and 0.50 for APRI).

**Table 3:** Results of the linear mixed-effects models.

Predictor	FIB-4		APRI	
	Model with no interaction	Model with the interaction term	Model with no interaction	Model with the interaction term
Diabetes (Yes *vs* No)	1.48 (1.21; 1.83) *P*<0.001	0.36 (0.09; 0.63) *P=*0.009	1.78 (1.38; 2.29) *P*<0.001	1.65 (1.18; 2.29) *P=*0.003
Time (Post Tx *vs* Pre Tx)	-0.30 (0.25; 0.35) *P*<0.001	0.29 (0.22; 0.37) *P*<0.001	0.24 (0.19; 0.29) *P*<0.001	0.22 (0.16; 0.30) *P*<0.001
Diabetes x Time	–	1.07 (0.74; 1.54) *P=*0.700	–	1.17 (0.76; 1.81) *P*=0.500
Age (Years)	1.02 (1.01; 1.03) *P=*0.001	1.02 (1.01; 1.03) *P*<0.001	1.00 (0.98; 1.01) *P*=0.500	1.00 (0.98; 1.01) *P*=0.500

Results are exponentiated beta coefficients representing ratios of geometric means.

APRI, Aspartate aminotransferase to platelet ratio index. FIB-4, Fibrosis Index Based on 4 Factors. Tx, Liver transplantation.

– is for "not applicable".

**Figure 1:**
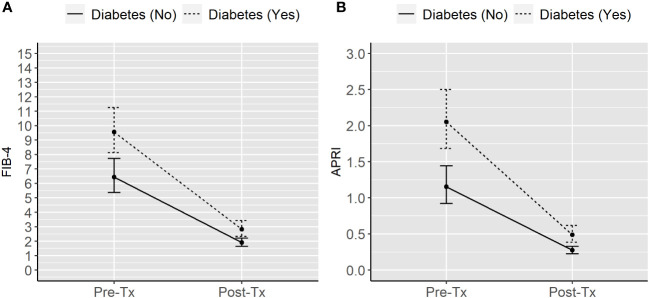
Model-based geometric means of FIB-4 **(A)** and APRI **(B)** in liver transplanted individuals according to diabetes status and time from transplantation. APRI, Aspartate aminotransferase to platelet ratio index. FIB-4, Fibrosis Index Based on 4 Factors. Tx, Liver transplantation.

**Figure 1** shows the model-based estimates of the geometric means of the two markers of liver fibrosis according to diabetes status and time from liver transplantation.

After liver transplantation, subjects with diabetes had higher fasting glycemia and HbA1c than non-diabetic individuals, while no significant differences were found for weight changes or other metabolic risk factors considered (**Table 4**).

**Table 4:** Metabolic risk factors post liver transplantation according to diabetes status.

Metabolic risk factor	Non-diabetic patients, Post-Tx, N = 72^1^	Patients with diabetes, Post-Tx, N = 39^1^	*P* value^2,3^
Weight change (kg)	1.2 (-4.2, 4.8)	2.0 (-1.5, 4.6)	0.568
BMI change (kg/m^2^)	0.1 (-1.7, 1.3)	0.7 (-0.4, 1.9)	0.206
Fasting glycemia (mg/dL)	92.0 (85.8, 99.5)	119.0 (101.5, 133.5)	<0.001
HbA1c (%)	5.2 (4.7, 5.7)	6.1 (5.6, 6.8)	<0.001
Serum creatinine (mg/dL)	1.0 (0.8, 1.2)	1.1 (1.0, 1.4)	0.200
Total cholesterol (mg/dL)	164.0 (144.0, 188.3)	161.0 (133.5, 193.5)	0.682
HDL cholesterol (mg/dL)	47.0 (40.8, 58.3)	41.0 (34.5, 53.5)	0.144
LDL cholesterol (mg/dL)	91.0 (71.5, 114.9)	94.0 (65.9, 122.0)	0.995
Triglycerides (mg/dL)	111.5 (85.8, 149.0)	115.0 (98.5, 146.5)	0.568

BMI, Body mass index. HbA1c, Glycated haemoglobin. HDL, High density lipoprotein. LDL, Low density lipoprotein.

^1^ Data are median (25^th^ – 75^th^ percentile).

^2^ Wilcoxon rank sum test.

^3^ False discovery rate correction for multiple testing.

## 4 Discussion

In our study, we recorded a high prevalence of diabetes in individuals with advanced hepatopathy who were candidates for liver transplantation and this condition was related to higher indices of liver fibrosis. Additionally, the study also found that after two years from liver transplantation the prevalence of diabetes remained elevated, with people with diabetes having a higher degree of liver fibrosis as compared to non-diabetic individuals.

The relationship between pre-transplant diabetes and a more advanced stage of fibrosis in subjects with hepatopathy has been previously demonstrated (34). However, the novelty of this study lies in the finding that subjects with diabetes continue to display elevated indicators of liver fibrosis two years after liver transplantation.

As mentioned before, diabetes is a condition frequently associated to liver cirrhosis. It is related to a worse outcome, due to increased mortality and more frequent complications of liver disease (22, 35, 36), although it’s not considered as a variable to assess the severity of liver disease in the most used staging and prognostic scores, as Child-Pugh and MELD.

Even after liver transplantation, the presence of glucose abnormalities is closely related to a worse prognosis, with higher risk of cardiovascular disease, liver rejection, infections and death (34, 37–39). Liver biopsy and FibroScan are actually the gold standard for assessing liver fibrosis, non-invasive methods as serum tests are gradually becoming more and more reproducible, available and accurate to detect liver fibrosis (9). In this context, FIB-4 and APRI have been demonstrated to be trustworthy as serum markers-based scores to assess liver fibrosis in subjects with hepatopathy from different aetiologies (15–17) and in liver-transplanted individuals (20, 40).

Activation of hepatic stellate cells has a crucial role in fibrosis development because of their extracellular matrix production during hepatic injury (41). Both genetic and environmental factors can impact on the pace of progression to cirrhosis. To date, the only established risk factor for developing new fibrosis after organ transplantation is the recurrence of the underlying hepatopathy such as viral hepatitis, primary biliary cirrhosis and primary sclerosing cholangitis (42–45).

To our knowledge, there are no studies assessing the presence of other pre and post-transplantation risk factors for developing new fibrosis after liver transplantation. For this reason, we performed this simple and reproducible evaluation on a population of subjects with diabetes referring to our Diabetes Center, to evaluate if diabetes could worsen liver fibrosis before transplantation or could represent a further risk factor for developing new fibrosis after surgery.

The novelty of our research is the demonstration that diabetes could also represent a potential risk factor for developing new fibrosis, assessed with FIB-4, after surgery, although the underlying pathogenetic mechanisms are still to be completely clarified.

A limitation of this study is the possible presence of NAFLD in the transplanted organ, as a potential confounding factor in the assessment of liver fibrosis in the post-transplant evaluation. As well as the presence of NAFLD in the transplanted organ, several variables, as age of both donor and recipient, therapeutic schemes used for immunosuppression and concomitant viral infections, may negatively impact on a possible recurrence of fibrosis after surgery (46).

Moreover, data from literature report a prevalence of 20% of *de novo* NAFLD in liver transplanted individuals, mostly due to the significant weight gain and the developing of metabolic syndrome following surgery (47). Despite this we, couldn’t investigate the presence of insulin resistance, as fasting insulin levels being not available in this population for calculation of HOMA index.

Again, a recent meta-analysis, aimed to evaluate the accuracy of non-invasive indices and FibroScan in detecting *de novo* hepatic fibrosis after liver transplantation, demonstrated a better prediction of recurrent fibrosis by transient elastography, if compared to APRI and FIB-4 scores in liver-transplanted individuals (48, 49). APRI and FIB-4 have been also used as prognostic tools in people who had hepatic transplantation and their trend overtime has been related to several long-term outcomes, as death and liver rejection.

Heterogeneity of cut-offs used in the different studies is one of the most critical limits for these non-invasive biomarkers, which may affect their effective reliability in real-world practice (50).

For this, the gold standard for diagnosis and management of liver fibrosis remains liver biopsy.

Finally, we aim to confirm the evidences we found in this study on a wider population and in a longer follow up period.

In conclusion, individuals with diabetes need a closer follow-up in order to promptly recognize people to refer to a hepatology unit for elastography and, if the recurrence of new fibrosis is confirmed, to undertake the adequate therapeutic measures aimed at limiting its possible complications.

## Data availability statement

The raw data supporting the conclusions of this article will be made available by the authors, without undue reservation.

## Ethics statement

The studies involving humans were approved by Comitato Etico Territoriale Lombardia 3. The studies were conducted in accordance with the local legislation and institutional requirements. The participants provided their written informed consent to participate in this study.

## Author contributions

VG: Conceptualization, Data curation, Writing – original draft, Writing – review & editing. IC: Writing – original draft. GA: Data curation, Formal Analysis, Methodology, Software, Writing – original draft. AG: Writing – original draft. SG: Writing – review & editing. MD: Writing – review & editing. EO: Supervision, Validation, Writing – review & editing. VR: Writing – review & editing.
